# Combination Therapies for Nonalcoholic Fatty Liver Disease

**DOI:** 10.3390/jpm12071166

**Published:** 2022-07-18

**Authors:** Evangelia S. Makri, Eleftheria Makri, Stergios A. Polyzos

**Affiliations:** First Laboratory of Pharmacology, School of Medicine, Aristotle University of Thessaloniki, 54124 Thessaloniki, Greece; msevange@auth.gr (E.S.M.); elemakste@auth.gr (E.M.)

**Keywords:** combination, insulin resistance, multifactorial pathogenesis, nonalcoholic fatty liver disease, nonalcoholic steatohepatitis, treatment

## Abstract

Nonalcoholic fatty liver disease (NAFLD) is considered a highly prevalent disease associated with various co-morbidities that lead to socioeconomic burden. Despite large-scale investigation, no pharmacological treatment has been approved specifically for NAFLD to date. Lifestyle modifications and diet are regarded as highly beneficial for the management of NAFLD, albeit with poor compliance, thus rendering pharmacological treatment highly important. Based on the current failure to discover a “magic bullet” to treat all patients with NAFLD and considering the multifaceted pathophysiology of the disease, combination therapies may be considered to be a rational alternative approach. In this regard, several drug categories have been considered, including, but not limited to, lipid-lowering, anti-hypertensive, glucose-lowering, anti-obesity, anti-oxidant, anti-inflammatory and anti-fibrotic medications. The aim of this review is, in addition to summarizing some of the multiple factors contributing to the pathophysiology of NAFLD, to focus on the efficacy of pharmacological combinations on the management of NAFLD. This may provide evidence for a more personalized treatment of patients with NAFLD in the future.

## 1. Introduction

Nonalcoholic fatty liver disease (NAFLD), a leading cause of chronic liver disease, represents a phenotypic spectrum, including simple steatosis or nonalcoholic fatty liver (NAFL), nonalcoholic steatohepatitis (NASH), which may progress to hepatic fibrosis, cirrhosis, and hepatocellular carcinoma, which may occur even in the absence of liver cirrhosis [1]. NAFLD is associated with hepatic and extrahepatic morbidity and mortality [2]. Cardiovascular disease (CVD) is the primary cause of death in patients with NAFLD, followed by malignancies, whereas hepatic diseases are the third cause of death [3,4]. There is a bidirectional association of NAFLD with metabolic syndrome (MetS), as reflected in the recently recommended change in the nomenclature to metabolic (dysfunction)-associated fatty liver disease (MAFLD) [5,6]; this recommendation, despite raising extensive discussion [7], also carries a change in the definition of the disease, highlighting its metabolic and multifactorial nature.

The worldwide prevalence of NAFLD is about 25%, with the highest rates in South America and Middle East and the lowest in Africa [8]. The prevalence of NAFLD is expected to further increase in the near future, thus adding to the personal and socioeconomic burden [9]. Nonetheless, an effective medication specifically for NAFLD has not been approved yet [10]. Although the treatment of NAFLD is a field of extensive research, a “magic bullet” to treat all NAFLD patients has not been discovered and probably will not be discovered, partly due to the multifactorial nature of the disease [11,12]. Thus, considering the multiple-hit pathogenesis of NAFLD, combination therapies, i.e., administering two or more medications that target different pathogenic factors, may represent a rational alternative therapeutic approach [12,13].

The aim of this review is, in addition to summarizing some of the multiple factors contributing to the pathophysiology of NAFLD, to focus on the efficacy of pharmacological combinations on the management of NAFLD. This may provide evidence for a more personalized treatment of patients with NAFLD in the future.

## 2. Major Contributors to the Pathophysiology of NAFLD

The proposed pathophysiological model of “multiple parallel hits” involves multiple pathogenic factors (“hits”) that act simultaneously and/or sequentially, including but not limited to lifestyle, environmental, genetic and epigenetic contributors [11]. Excessive fat is stored intra-hepatically due to insulin resistance (IR)-driven increased lipolysis in the adipose tissue and hepatic *de novo* lipogenesis [14,15]. Furthermore, adipose tissue dysfunction, the dysbiosis of gut microbiota, lipotoxicity, oxidative stress and inflammasome activation may also contribute to NAFL, but also to its progression to NASH [11,16]. If factors contributing to this low-grade but chronic hepatic inflammation are not sufficiently and timely managed, then the disease may progress to hepatic fibrosis, which is regarded as the main histological prognostic factor of advanced disease [17,18]. Importantly, different pathogenic factors may contribute to periods of different duration in the affected individuals, thus rendering NAFLD a highly heterogenous disease [17].

The main pathogenic contributors to NAFLD are summarized herein, in order to support that the multifactorial nature of the disease may require combination treatment, possibly on a personalized basis.

### 2.1. Genetic Factors

There are several genetic variants implicated in the hepatic lipid metabolism, thus contributing to NAFLD. The better-established genetic associate of NAFLD is the single-nucleotide polymorphism rs738409 of the patatin-like phospholipase domain-containing protein 3 (PNPLA3) gene [19,20]. Other genetic variations having also been linked with NAFLD, including, but not limited to, transmembrane 6 superfamily member 2 (TM6SF2), membrane-bound O-acyltransferase domain-containing 7 (MBOAT7), and glucokinase regulator (GCKR) genes [21]. As genetic polymorphisms as well as epigenetic modifications have been associated with oxidative stress, hepatic inflammation, and fibrosis in NAFLD, further evaluation of their cross-talk with the disease may provide new targets and a more personalized approach to the management of NAFLD in the future, e.g., individualized genetic therapy [22]. Additionally, genes that influence pathways of IR and liver fat accumulation may possibly affect NAFLD progression; thus, an in-depth analysis of the genetic and epigenetic alterations could not only enhance our knowledge on the pathogenesis of the disease, but may also provide multiple therapeutic choices [21].

### 2.2. Intrahepatic Lipid Accumulation

Excessive fat is deposited intra-hepatically, mainly as triglycerides (TGs) [23]. In NAFLD, this mainly derived from the increased liver uptake of free fatty acids (FFAs), which are estimated to be derived approximately: (a) 60% from adipose tissue lipolysis; (b) 25% from hepatic *de novo* lipogenesis due to the increased transformation of other substrates (mainly carbohydrates, e.g., fructose) to FFAs; and (c) 15% from diet [24]. In the liver, FFAs may be esterified to TGs and stored, or β-oxidized in the mitochondria and peroxisomes to produce energy [25]. The low export of FFAs, as a result of the impaired secretion of very low-density lipoprotein cholesterol (VLDL-C), may also contribute to intra-hepatic fat accumulation [26].

Emerging evidence underlines *de novo* lipogenesis as an important driver of NAFLD. Key transcription factors in the *de novo* lipogenesis are the sterol regulatory element-binding protein (SREBP)-1, the carbohydrate response element-binding protein (ChREBP) and the peroxisome proliferator-activated receptor (PPAR)-γ [27]. SREBP-1c is regulated by insulin through mechanisms that involve the liver X receptor (LXR)α [25]. ChREBP also interferes with LXRs, but it is mainly activated directly by carbohydrates, such as glucose and fructose [25,28]. In addition, PPARs along with PPAR-γ coactivator-1 (PGC-1) cross-talk with the two above-mentioned transcription factors and contribute to the orchestration of the intra-hepatic regulation of carbohydrate and lipid metabolism [29]. Of note, fructose intake is metabolized without regulation by insulin, leading to ATP depletion and oxidative stress, followed by organelle dysfunction [28].

### 2.3. Mitochondrial Dysfunction and Endoplasmic Reticulum Stress

Lipotoxicity and glucotoxicity contribute to hepatotoxicity via mitochondrial damage, the endoplasmic reticulum (ER) stress reaction, and the activation of the cell death pathway [30]. In particular, oxidative stress increases through ER damage, thus provoking a vicious cycle between oxidative stress and ER damage. The excess saturated fatty acids (FAs), hyperglycemia and by-products impair the function of ER, promoting the pathway of unfolded protein response (UPR). Under normal circumstances, UPR resolves misfolded proteins and regulates ER homeostasis. On the contrary, if UPR is impaired, hepatic inflammation and apoptosis are activated through the activation of Jun N-terminal kinase (JNK) and hepatic steatosis is aggravated through SREBP-1c pathway activation [31]. As a consequence, the dysfunction of intracellular organelles and cell damage may occur that may lead to hepatocellular damage and apoptosis [11]. Additionally, reactive oxygen species (ROS) generation oxidizes lipid deposits, leading to lipid peroxidation and, consequently, mitochondrial DNA damage and the depletion of protective antioxidant mechanisms [32]. With the above considered, mitochondrial dysfunction and ER play key roles in the pathogenesis of NAFLD.

### 2.4. Adipose Tissue Dysfunction

A Western diet, rich in saturated fat and simple, processed carbohydrates, together with a sedentary lifestyle, increases IR [33]. Normally, adipose tissue secretes adipokines and cytokines, mainly produced by the adipocytes and immune cells infiltrating adipose tissue, respectively [17]. At a state of positive energy balance, adipose tissue expands, thus causing alterations in adipokine secretion. Specifically, beneficial insulin-sensitizing and anti-inflammatory adipokines/cytokines, such as adiponectin and interleukin (IL)-10, are decreased, while pro-inflammatory adipokines/cytokines, including leptin, resistin, IL-1β, IL-6, and IL-8, are increased [34,35]. This imbalance results in the infiltration of adipose tissue by more immune cells, thus aggravating the low-grade but chronic inflammation of adipose tissue, which cross-talks with the above-mentioned low-grade, chronic inflammation of the liver [36]. Thus, adipokine/cytokine dysregulation may contribute to NAFL, NASH and possibly to advanced forms of the disease.

### 2.5. Gut Microbiota Dysbiosis

Gut microbiota can be affected by environmental factors, e.g., dietary changes and antibiotics, as well as genetic predisposition, resulting in the dysbiosis of the microbiota, which is associated with the accumulation of short-chain FAs and lipopolysaccharides [17,37]. When polysaccharides are fermented into monosaccharides and short-chain FAs, their absorption is facilitated [11]. Dietary imbalance may also lead to the disruption of the gut barrier, bacterial translocation, and Toll-like receptor-induced inflammation [11,38]. These changes further facilitate the influx of the metabolites of microbiota to the systemic circulation, thus possibly affecting other organs, including the liver (the so-called gut–liver axis), in which they may contribute to the development and progression of NAFLD [17,37].

### 2.6. Other Pathogenic Contributors

Apart from those mentioned above, there are more established contributors to the pathogenesis of NAFLD; there are other potential contributors, including, but not limited to, metabolic and endocrine factors (e.g., low thyroid hormone, growth hormone or sex hormone concentrations, hypercortisolemia, iron overload), infections (e.g., Helicobacter pylori, COVID-19); and endocrine disruptors [39,40,41,42]. Considering all of the above, a better understanding of the complicated pathophysiology of NAFLD and the interplay among distinct established and potential contributors may lead to the more efficient management of this multiple-hit disease.

## 3. Combination Treatment of NAFLD

**Figure 1:**
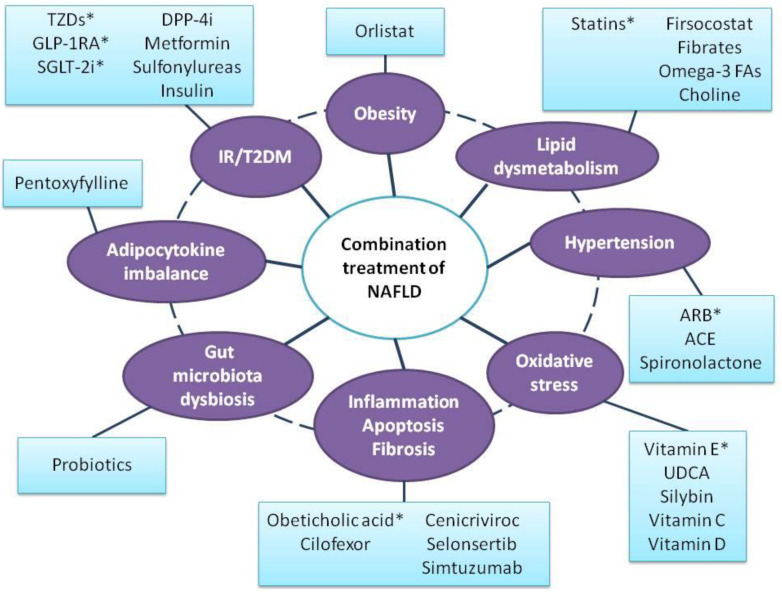
The multifactorial pathophysiology of NAFLD with possible medications investigated in combination. NAFLD has been characterized as a “multiple-hit” disease. Lipid dysmetabolism, insulin resistance, adipocytokine dysregulation, gut–liver axis dysfunction, oxidative stress and genetic predisposition are considered the main factors that trigger hepatic steatosis and the progression to inflammation and fibrosis. In parallel to these contributors, several co-morbidities such as obesity, T2DM, dyslipidemia and hypertension, lie in parallel with NAFLD, leading to increased morbidity and mortality. The multifaceted pathogenesis of the disease and the failure of current monotherapies to provide a definite solution to the management of NAFLD may inspire a shift of research towards combined therapies. Each category indicates all potential medications that have been used in combination with at least another one medication within the same or another category. Medications highlighted with an asterisk (*) have provided more favorable results and may be more eligible for future research with combination treatment in NAFLD. It is highlighted that some medications may act with more than one mechanism. Abbreviations: ACE—angiotensin-converting enzyme; ARB—angiotensin receptor blocker; DPP-4i—dipeptidyl peptidase-4 inhibitors; FAs—fatty acids; GLP-1RA—glucagon-like peptide-1 receptor agonists; IR—insulin resistance; SGLT-2i—sodium glucose cotransporter-2 inhibitors; TZDs—thiazolidinediones; T2DM—type 2 diabetes mellitus; UDCA—ursodeoxycholic acid.

Although the treatment of NAFLD is a hot topic, there is no approved medication for this highly prevalent disease [10]. The failure of various monotherapies and the completion of clinical trials without meeting the primary outcome(s) may highlight that there is not a “magic bullet” to treat all patients with NAFLD, at least partly due to the high heterogeneity of the pathogenic contributors. Therefore, it seems to be rational that targeting more than one pathogenic contributor of the disease may be a more efficient approach [12,13]. For example, instead of targeting IR or oxidative stress, targeting both may be more beneficial. In this regard, there are several published clinical trials investigating the efficacy of combination therapy on NAFLD (Table 1) whose results are discussed herein, as well as ongoing studies (Table 2). The main targets of the medications used in combination treatments for NAFLD are summarized in Figure 1.

### 3.1. Anti-Obesity Medications

Obesity constitutes a major driver of the development and progression of NAFLD; in line, weight reduction is the cornerstone of the management of NAFLD [15]. In this regard, orlistat, a reversible inhibitor of gut and pancreatic lipase which hinders the absorption of a part of dietary TGs, is approved for the management of obesity and decreased liver function tests (LFTs) in cases of weight loss; however, histological improvement was not consistent [15]. In a randomized controlled trial (RCT) with overweight individuals with histologically confirmed NASH, there were no differences regarding histological and biochemical outcomes between the group that received orlistat and vitamin E vs. the group of orlistat monotherapy [43], i.e., vitamin E did not have an additive effect on any effect of orlistat.

### 3.2. Ursodeoxycholic Acid

Ursodeoxycholic acid (UDCA) is a secondary bile acid which is primarily produced by intestinal microbiota. UDCA has been shown to be effective for cholestatic disorders, although was rather neutral in clinical studies with NAFLD patients [10]. Combination therapies of UDCA with vitamin E, tiopronin, polyene phosphatidylcholine, silymarin and glycyrrhizin showed greater improvements in LFTs than the respective monotherapies [74]. The majority of studies have evaluated the combination of UDCA with vitamin E (Table 1). Dufour et al. evaluated UDCA and vitamin E vs. UDCA and placebo vs. placebo/placebo in patients with biopsy-proven NASH [44]. A 2-year histological evaluation revealed a decrease in steatosis in the UDCA/vitamin E group, whereas there was no significant change in the monotherapy group. However, inflammation and fibrosis were not improved and there were no differences between the three groups concerning histological assessment, thus rendering the additive effect of vitamin E to UDCA questionable. Another study investigating the long-term efficacy of UDCA and vitamin E demonstrated a decrease in LFTs (aspartate aminotransferase (AST), alanine aminotranferase (ALT) and γ-glutamyl transferase (γ-GT)) [45]. Nonetheless, repeat biopsy was performed in only 10 patients (10%) after a median duration of 5 years from the initiation of treatment: NAFLD activity score (NAS) was improved in seven patients and the mean change in score was −1.0. Similar results for LFTs were obtained in a retrospective comparative study that divided patients with a histological confirmation of NAFLD into three groups: group I was subjected only to lifestyle counseling, group II to vitamin E and lifestyle counseling and group III to UDCA, vitamin E, and lifestyle counseling [46]. Higher rates of patients with LFT normalization were observed in the combination group; however, the difference in ALT was significant only between group 1 and 3 in the pairwise comparisons [46]. Considering the above, current data do not favor the administration of UDCA as monotherapy or in combination with vitamin E in patients with NASH.

### 3.3. Farnesoid X Receptor Agonists

Farnesoid X receptor (FXR) is a nuclear receptor, expressed in the liver and intestine, which regulates various metabolic pathways, including bile acid synthesis, glucose, and lipid homeostasis [75]. Obeticholic acid, the first FXR agonist investigated as a monotherapy for NASH, provided favorable results in hepatic histology, including an improvement in fibrosis [76]. Cilofexor, another FXR agonist, is currently being investigated for the treatment of NASH with a beneficial effect on steatosis [77]. Pruritus was a common adverse effect in the group receiving a high dose of cilofexor, as in the case of obeticholic acid [76]. In this case, combination therapy may reduce the dose of cilofexor, thus reducing the possibility of pruritus, and may simultaneously have an additive effect on hepatic histology. In this regard, cilofexor was evaluated in combination with selonsertib and firsocostat in an ATLAS trial, as described in more detail below [47].

### 3.4. Fatty Acid Synthesis Enzyme Inhibitors

Acetyl-CoA carboxylase (ACC) constitutes a key enzyme of *de novo* lipogenesis and firsocostat—an inhibitor of ACC—reduced the hepatic steatosis and serum biomarkers of fibrosis in a phase 2 RCT [78,79]. Based on these observations, firsocostat was evaluated in combination with selonsertib and cilofexor in an ATLAS trial, as described in more detail below [47].

### 3.5. Anti-Apoptotic Medications

Since apoptosis is implicated in the pathogenesis of NAFLD and seems to be related to fibrosis, the apoptosis signal-regulating kinase (ASK)1 inhibitor selonsertib is regarded as a medication with possible anti-inflammatory and anti-fibrotic effects; however, selonsertib monotherapy failed to meet the primary endpoint of clinical trials including patients with NASH and fibrosis stage F3/F4 [80]. Subsequently, selonsertib was evaluated in combination with firsocostat and cilofexor in an ATLAS trial, which is a phase 2b RCT for patients with NASH and F3/F4 [47]. Patients were allocated to receive placebo or monotherapy with selonsertib, cilofexor, firsocostat or combination therapy with all possible pairs of combinations of the referred medications for one year [47]. Improvement in fibrosis without the worsening of NASH (primary endpoint) was not changed between the treatment groups and the placebo. However, the percentage of patients with ≥2-point improvement in NAS was higher in cilofexor/firsocostat compared with the placebo group. Moreover, progression to cirrhosis was less common in cilofexor/selonsertib than in the placebo group.

Selonsertib was also tested in a phase 2 RCT in combination with simtuzumab, a humanized monoclonal antibody against the formation of collagen chemical bonds, regarded as an anti-fibrotic medication [48]. NASH patients with fibrosis stage F2/F3 received selonsertib or simtuzumab alone or in combination to show that the addition of simtuzumab did not add to any anti-fibrotic effect of selonsertib [48].

### 3.6. Anti-Oxidant Medications

Among antioxidant medications, vitamin E has been proposed as an off-label treatment in selected patients with NASH and F ≥ 2 [81], improving LFTs and hepatic histology, albeit not hepatic fibrosis [82]. However, the increased risk of cardiovascular adverse effects and prostate cancer (in men) hinders the widespread use of high dose (800 IU/d) vitamin E in the long term [83].

Vitamin E has been investigated with other medications in several trials. In a double-blind RCT, NASH patients were administered a combination of vitamin E and vitamin C or placebo and they were followed-up for 6 months [49]. There were no differences between the two groups concerning hepatic inflammation and fibrosis. However, the design of this study could not show an additive effect of the combination vs. monotherapy, since there were no groups with monotherapies.

The combination of vitamin E with vitamin C vs. placebo was also investigated in a pediatric population with biopsy-proven NAFLD [50]. After 24 months, any improvement in LFTs and histological findings (steatosis, lobular inflammation and ballooning) were similar between groups. Thus, an additive effect of vitamin C to that of vitamin E was not shown.

In a 6-month RCT, vitamin E was also combined with vitamin D and silybin, an antioxidant silymarin extract of milk thistle, and the combination was compared with a group receiving no treatment for six months [51]. LFTs and hepatic steatosis (evaluated with transient elastography (TE)) were improved in patients assigned in the treatment group, but not to untreated patients. Again, a limitation of this study was the lack of groups with monotherapy.

Silybin was also used in a phytosome complex with phospatidylcholine in combination with vitamin E [52]. Biopsy-proven NAFLD patients received a combined treatment or placebo for 1 year. Although the ultrasonographic evidence of hepatic steatosis was similar between groups, hepatic steatosis, inflammation and, reportedly, fibrosis were improved in the treatment group within a subset of patients (*n* = 32) subjected to liver biopsy at 12 months. Similarly to the above two studies, the additive effect of silybin/phospatidylcholine on vitamin E could not be shown in this study.

### 3.7. Hypolipidemic Medications

Lipid-lowering medications have been extensively investigated in patients with NAFLD, who are regarded as a high-risk population for CVD [3]. Statins play a key role in the treatment of dyslipidemia and have been proposed for patients with NAFLD and dyslipidemia, although data regarding hepatic histology are few and controversial [84].

Regarding combination treatment, data from an open-label RCT in NAFLD patients showed that the rates of patients with NAFLD resolution were higher in the groups of atorvastatin monotherapy and the combination of atorvastatin/fenofibrate compared with fenofibrate monotherapy [53]. This implies that fenofibrate has no additive effect on improvements in hepatic steatosis. Atorvastatin was also combined with vitamin E and vitamin C to lead to lower rates of NAFLD in the treatment group compared to placebo [54]. However, this study cannot show whether the combination treatment was superior to either monotherapy, owing to the lack of the relevant groups of monotherapy.

Concerning polyunsaturated FAs, such as omega-3 FAs, their effectiveness on NAFLD as monotherapy seems to be neutral, although their use is recommended to treat hypertriglyceridemia in NAFLD patients, similarly to non-NAFLD individuals [85]. However, omega-3 FAs have been used in combination with other medication in clinical trials with pediatric NAFLD. Docosahexaenoic acid (DHA), a highly unsaturated omega-3 FA, was administered in combination with vitamin D in an RCT with obese NAFLD children [55]. The combination treatment reportedly improved LFTs, hepatic steatosis and inflammation, but not fibrosis [55]. DHA was also investigated in children with biopsy-proven NASH in combination with choline and vitamin E vs. placebo [56]. After 12 months of treatment, LFTs, hepatic steatosis and inflammation, but not fibrosis, were decreased only in the combination group. However, the design of both these studies cannot show an additive effect of DHA on pediatric NAFLD.

### 3.8. Mineralocorticoid Receptor Antagonists

By acting on the mineralocorticoid receptors of the liver, aldosterone induces the expression of several collagen genes, activates genes controlling tissue growth factors, such as transforming growth factor (TGF)-β and plasminogen activator inhibitor type 1, and induces the expression of genes mediating inflammation [86]. Collectively, the actions of aldosterone in the liver favor inflammation and fibrosis. A mineralocorticoid receptor antagonist, spironolactone, has been investigated in mouse models and has shown improvements in liver steatosis and the suppression of lipogenic genes and proinflammatory cytokines [87]. In an RCT, the treatment of NAFLD patients with spironolactone in combination with vitamin E improved IR more than vitamin E monotherapy at two months and decreased NAFLD liver fat score, an index of hepatic steatosis, more than vitamin E monotherapy after one year of treatment [57,88]. This study may warrant larger studies with paired liver biopsies to show an additive effect of spironolactone to vitamin E, especially in terms of hepatic fibrosis.

### 3.9. Anti-Diabetic Medications

Certain medications approved for the treatment of T2DM have been evaluated or are under evaluation in combination with others for the treatment of NAFLD. Pioglitazone is a PPAR-γ agonist belonging to the class of thiazolidinediones [89]. Pioglitazone ameliorates hepatic steatosis and inflammation, although its effect on fibrosis is marginal [89,90]. As mentioned above for vitamin E, pioglitazone has also been recommended for the off-label treatment of NASH with F ≥ 2 by most guidelines [81]. The main contraindications of pioglitazone are the coexistence of advanced heart failure, bladder cancer and osteoporosis [81]. Rosiglitazone is another thiazolidinedionic PPAR-γ agonist shown to decrease hepatic steatosis, but not inflammation or fibrosis [91]; however, the use of rosiglitazone has been restricted because of concerns about increasing the cardiovascular risk [89].

The combination of pioglitazone and vitamin E was compared with vitamin E monotherapy in a 6-month RCT in patients with NASH [58]. The improvement in inflammation was greater in the combination group, whereas changes in LFTs and fibrosis were similar between groups. However, the sample of this study may have been small and the duration short (Table 1) to draw definite conclusions, especially for endpoints such as hepatic fibrosis.

Combining a statin with pioglitazone has also been proposed to manage NAFLD and decrease the related cardiovascular risk [92]. However, this combination has not been investigated yet and only a case report has demonstrated the amelioration of LFTs and ultrasonographic hepatic steatosis in a 47-year man with NAFLD receiving rosuvastatin and pioglitazone for 9 months [59]. Although definite conclusions could not be made by a case report, we favor the set of clinical studies examining the combined effects of pioglitazone (or other PPAR-γ agonists) in combination with a statin in NASH patients.

Pioglitazone was also evaluated in combination with insulin in two RCTs [60,61]. One of them did not reveal a significant improvement in hepatic fat evaluated with CT, either in the insulin/pioglitazone group or in the insulin/placebo group. However, the duration of the study was relatively short (3–4 months). In the other study, the combination of pioglitazone and insulin, but not insulin monotherapy, decreased hepatic steatosis, as evaluated with magnetic resonance spectroscopy (MRS) after 6 months of treatment. However, the between-group comparison was not significant [61]. Based on the results of these studies, the addition of pioglitazone to insulin does not seem to have an additive benefit on NAFLD.

Regarding rosiglitazone, in an open-label RCT with biopsy-proven NASH patients, rosiglitazone was administered alone or combined with metformin or losartan [62]. Metformin is a first line anti-diabetic medication with a limited effect on NAFLD, despite targeting IR [10]. Losartan, an angiotensin II receptor blocker, approved for the treatment of arterial hypertension, showed promising results as a monotherapy in a small study [93]. Since there were no histological differences between the rosiglitazone monotherapy, rosiglitazone/metformin or rosiglitazone/losartan groups [62], the results did not favor rosiglitazone and metformin or rosiglitazone and losartan over rosiglitazone monotherapy for the management of NAFLD. According to a second RCT evaluating NASH patients with NAS ≥ 5, NAS was improved in rosiglitazone and in the combination group (rosiglitazone plus metformin), with no improvement in the metformin monotherapy group, but the effect on fibrosis was not significant in any of the groups [63]. Consequently, metformin does not seem to offer additional benefits in terms of NAFLD when added to rosiglitazone.

Metformin was also investigated in a small open-label clinical trial in combination with insulin vs. in combination with pioglitazone and glyburide (a sulfonylurea approved for the treatment of T2DM) [64]. At the end of the study, there was no superiority of one group over the other in terms of hepatic steatosis, as evaluated with MRS. The combination of another sulfonylurea (glibenclamide) vs. the combination of glibenclamide with another thiazolidinedione (troglitazone) was also investigated [65]. The addition of troglitazone to glibenclamide decreased LFTs and hepatic steatosis as compared with glibenclamide monotherapy [65]. Nonetheless, it should be noted that troglitazone has been withdrawn due to rare but severe events of hepatic failure [89].

Pentoxifylline is a xanthine derivative used for the management of peripheral vascular disease that has also been investigated in NAFLD, owing to its reportedly anti-TNF properties [94]. However, when the combination of metformin and pentoxifylline was compared with no treatment in a pilot, one-year RCT, no effect was observed on hepatic histology [66].

Incretin-based therapies, including glucagon-like peptide-1 receptor agonists (GLP-1RAs) and dipeptidyl peptidase-4 (DPP)-4 inhibitors (DPP-4i), are licensed anti-diabetic medications that have also been investigated for the management of NAFLD. GLP-1RA increase insulin secretion by stimulating β-cells in response to glucose presence and DPP-4i inhibit DPP-4, an enzyme catalyzing the proteolytic degradation of endogenous GLP-1, thus prolonging the action of GLP-1 [95,96]. Incretin-based agents have been investigated in patients with NAFLD and seem to decrease LFTs [96]. Although GLP-1RA seem to have favorable outcomes, even resulting in the histological resolution of NASH in some cases, DPP-4i have shown minimal to null effects on NAFLD [95,97,98]. Sodium glucose cotransporter-2 inhibitors (SGLT-2i) are another class of approved anti-diabetic medications that inhibit renal glucose reabsorption, thus leading to glucose control and weight reduction, and thus also favoring NAFLD patients [99]. Although SGLT-2i showed encouraging results in improving LFTs and hepatic steatosis, more studies with repeat liver biopsies are needed, especially for the evaluation of their effect on fibrosis [99]. SGLT-2i and GLP-1RA also seem to lower all-cause and cardiovascular mortality, non-fatal myocardial infarction, and kidney failure [100]. Given that CVD is the first cause of death in patients with NAFLD [101], as mentioned above, these medications may prove essential for the management of NAFLD and its associated cardiovascular risk [4]; thus, studies investigating the combination of GLP-1RA and SGLT-2i in NAFLD patients are warranted.

Exenatide, a GLP-1RA, was administered in combination with pioglitazone vs. pioglitazone monotherapy and showed a greater reduction in LFTs and hepatic steatosis in the combination group [67]. Exenatide was also evaluated as an add-on treatment to insulin glargine to show a greater reduction in LFTs and hepatic steatosis, as compared with the combination of insulin aspart and insulin glargine [68]. However, studies with histological confirmation are needed.

On the contrary, when exenatide was investigated in combination with dapagliflozin (a SGLT-2i) vs. dapagliflozin and a placebo in a 24-week RCT, no difference in hepatic steatosis, measured with MRS, was observed between groups [69]. In a *post hoc* analysis of the “DURATION-8” RCT, the combination of dapagliflozin and exenatide was compared vs. either monotherapy plus a placebo to show that the combination led to greater improvement in noninvasive indices of hepatic steatosis (fatty liver index (FLI) and NAFLD liver fat score) than both monotherapies [70]. Contrary to the steatosis markers, changes in fibrosis noninvasive indices (NAFLD fibrosis score and FIB-4) were not different between groups. These results may be encouraging, but there is need for studies with repeat liver biopsies specifically designed towards this aim.

Dapagliflozin was also evaluated in combination with omega-3 FAs vs. either monotherapy vs. placebo in NAFLD patients with T2DM for 3 months (EFFECT-II RCT) [71]. Only the combination treatment improved hepatic steatosis, as evaluated with MRI–proton density fat fraction (MRI-PDFF) vs. placebo. However, an additive effect of omega-3 FA to dapagliflozin was not shown in this study. Moreover, dapagliflozin was compared with empagliflozin as an add-on treatment in patients with T2DM already receiving metformin, glimepiride and DPP-4i. However, between-group comparison did not demonstrate significant changes in LFTs [72].

Sitagliptin (a DPP-4i) in combination with metformin was also compared with the combination of metformin and glipizide (a sulfonylurea), the former showing greater effectiveness in reducing LFTs and hepatic steatosis [73]. However, studies with histological endpoints are needed to draw definite conclusions.

## 4. Closing Remarks

NAFLD is a highly prevalent disease with considerable morbidity and mortality, but without any approved medication to date, despite extensive research in the field [10]. Lifestyle modifications (diet and exercise) are considered the cornerstone for the management of NAFLD, but they are targets that are difficult to achieve and even more difficult to sustain in the long term [33]. Most medications evaluated to date failed to meet their primary endpoints; even if the endpoints were met referring to the mean, a considerable proportion of patients did not experience histological improvement, which may be partly attributed to the heterogenous pathogenesis of the disease [10]. As mentioned above, “multiple-hit” pathogenesis implies that multiple factors contribute to the pathogenesis of different patients. Furthermore, the strength of each contributor and its duration of action may also vary on an individual basis, further implicating the heterogeneity of the disease. This may render the need for combination treatment important, even in a personalized approach, after previous identification of the main pathogenic contributors to each specific patient [12,13]. For example, for an obese individual with NASH and dyslipidemia at high cardiovascular risk, we may provide orlistat (or a SGLT-2i) and a statin, but we should possibly avoid vitamin E, especially for a duration longer than 2 years. On the contrary, in a normoglycemic, normolipidemic lean individual with NASH at lower cardiovascular risk, orlistat, SGLT-2i or a statin may possibly have minimal or null effect. This patient may benefit from treatment with vitamin E in combination with obeticholic acid. We have also proposed a diabetes-like approach to manage comorbidities in NAFLD patients, i.e., obesity, T2DM, arterial hypertension, dyslipidemia, obesity, and CVD [12,13]. However, this approach, although seemingly rational, remains to be definitely proven.

A main target of the combination treatment is to increase the efficacy of monotherapy; however, another target of adding a second medication may be to alleviate the potential adverse effects of the first medication. A representative example is the addition of a statin to obeticholic acid, a FXR agonist, which has shown promising histological results in NASH patients without cirrhosis [102,103]. Pruritus, elevated low-density lipoprotein cholesterol (LDL-C), and decreased high-density lipoprotein cholesterol (HDL-C) were common adverse effects in NASH patients receiving obeticholic acid [76] and the co-administration of atorvastatin attenuated the elevation of LDL-C [104], although HDL-C levels were not increased. Specifically, in the case of NASH patients, increasing LDL-C may importantly affect cardiovascular morbidity and mortality; thus, attenuating this adverse effect may be important, although this remains to be shown [105]. Furthermore, the combination of obeticholic acid or cilofexor with an anti-histaminic medication may decrease the possibility of pruritus, thus increasing the adherence to treatment.

Existing combination therapies (Table 1) cover a variety of drug classes, such as antioxidant (vitamins, silybin), cytoprotective (UDCA), hypolipidemic (statins, omega-3 FAs), antidiabetic (thiazolidinediones, metformin, insulin, GLP-1RA, SGLT-2i), antihypertensive (spironolactone, losartan), anti-obesity (orlistat), anti-apoptotic, and anti-fibrotic (selonsertib, simtuzumab) agents. In general, most of the existing studies are limited by the lack of repeat liver biopsies, the small sample sizes, and their design, e.g., the lack of appropriate monotherapy groups, so as to show an additive effect of combination therapies vs. monotherapies. Furthermore, the different endpoints among different studies and the use of different diagnostic modalities for the endpoints render the comparative interpretation and indirect comparisons among them puzzled.

Ongoing studies (Table 2) aim to investigate additional and even more complex classes of medications, such as FXR agonists (tropifexor, MET409), PPAR-α and PPAR-γ agonists (saroglitazar), chemokine receptor 2 and 5 antagonists (cenicriviroc), bile acid metabolism-related substances (elobixibat, cholestyramine) and the inhibitor of leukotriene A4 hydrolase, the final enzyme in the synthesis of pro-inflammatory leukotriene B4 (LYS006).

It should be highlighted that the results of most of the studies included in our previous list of ongoing clinical trials on combination therapies, approximately 10 years ago, have not been published [12]. This is regarded as an important limitation, since the dissemination of even negative results of clinical trials would have been important to guide other researchers worldwide to “invest” their effort, time and resources in more appropriate directions. Even if a research project is terminated prematurely, the reasons for this premature discontinuation are important and should be announced. Last but not least, there is need to design the studies of NAFLD treatment based on standardized criteria and histological outcomes so that findings follow a common “language”; this may facilitate their interpretation, their comparison with each other, and their translation in clinical practice [106].

In conclusion, it seems that a “magic bullet”, i.e., “a one pill fits all” approach for patients with NAFLD does not exist and is difficult to be discovered, partly owing to the highly heterogenous pathogenesis of the disease. In this regard, combined therapies may target more than one pathogenic contributor (“hit”) of the disease simultaneously, which seems to be an appealing concept. Combination therapies may also pursue the same target, i.e., hepatic fibrosis and, in this regard, they may have additive or even synergistic effects on the target. Furthermore, the addition of a medication may allow a decrease in the dose of the other one which, thus, may be safer. Notably, the addition of a medication may also attenuate the adverse effects of an otherwise effective medication, e.g., the addition of a statin to obeticholic acid to attenuate an increase in LDL-C. Even more importantly, pathogenic contributors should be evaluated on an individual basis so as to target multiple contributors in personalized approaches.

## Figures and Tables

**Table 1:** Clinical studies having evaluated combination therapies in patients with NAFLD.

First Author, Year [Reference] ^1^	Groups (N)	Patients’ Characteristics	Study Type; Duration (Weeks)	Change in LFTs (Within Combination Group)	Change in Steatosis (Within Combination Group)	Change in Inflammation (Within Combination Group)	Change in Fibrosis (Within Combination Group)	Change in Additional Parameters	Between-Group Difference(s)
Harrison, 2009 [43]	(1) Vitamin E 800 IU (18) vs. (2) orlistat 360 mg + vitamin E 800 IU (23)	Overweight biopsy-proven NASH patients	RCT; 36	Yes (ALT, AST)	Yes (hepatic biopsy), only in the subgroup with weight lost ≥5%	Yes, only in the subgroup with weight lost ≥9%	No	NAS improvement, only in the subgroup with weight lost ≥9%	No
Dufour, 2006 [44]	(1) Placebo + placebo (15) vs. (2) UDCA 12–15 mg/kg + placebo (18) vs. (3) UDCA 12–15 mg/kg + vitamin E 800 IU (15)	Biopsy-proven NASH patients	RCT; 96	Yes (ALT, AST)	Yes (hepatic biopsy)	No	No	-	ALT decrease in group 3 vs. groups 1 and 2; AST decrease in group 3 vs. group 1
Pietu, 2012 [45]	(1) UDCA 1680 mg + vitamin E 555 IU (101)	Biopsy-proven NASH patients	Retrospective uncontrolled study; 192	Yes (ALT, AST, γ-GT)	Yes (hepatic biopsy) in 3/10 patients	Yes (hepatic biopsy) in 3/10 patients	Yes (hepatic biopsy) in 4/10 patients	NAS improvement in 7/10 patients	Νo control group
Madan, 2005 [46]	(1) Lifestyle counseling (18) vs. (2) lifestyle counseling + UDCA 600 mg (12) vs. (3) lifestyle counseling + UDCA 600 mg + vitamin E 400 mg (12)	Biopsy-proven NAFLD patients	Retrospective comparative study; 24	Yes (ALT, AST)	NA	NA	NA	-	ALT decrease in group 3 vs. group 1; higher percentage of patients normalized transaminases in group 3 vs. group 1 and 2
Loomba, 2021 [47]	(1) Placebo (39) vs. (2) selonsertib 18 mg (39) vs. (3) cilofexor 30 mg (40) vs. (4) firsocostat 20 mg (40) vs. (5) cilofexor 30 mg + selonsertib 18 mg (79) vs. (6) firsocostat 20 mg + selonsertib 18 mg (77) vs. (7) cilofexor 30 mg + firsocostat 20 mg (78)	Biopsy-proven NASH patients with F3 or F4	RCT; 48	NA	ΝA	NA	NA	-	ALT decrease in group 7 vs. group 1; steatosis, inflammation and NAS improved in group 7 vs. group 1 (hepatic biopsy)
Loomba, 2018 [48]	(1) Selonsertib 6 mg (20) vs. (2) selonsertib 18 mg (22) vs. (3) simtuzumab 125 mg (10) vs. (4) selonsertib 6 mg + simtuzumab 125 mg (10) vs. (5) selonsertib 18 mg + simtuzumab 125 mg (10)	Biopsy-proven NASH patients with F2 or F3	Open-label RCT; 24	NA	NA	NA	Yes (hepatic biopsy) in 4/10 patients (group 4) and in 2/9 patients (group 5)	-	NA
Harrison, 2003 [49]	(1) Placebo (22) vs. (2) vitamin E 1000 IU + vitamin C 1000 mg (23)	Biopsy-proven NASH patients	RCT; 24	No	NA	No	Yes (hepatic biopsy)	-	No
Nobili, 2008 [50]	(1) Placebo (28) vs. (2) vitamin E 600 IU + vitamin C 500 mg (25)	Biopsy-proven NAFLD children	Open-label RCT; 96	Yes (ALT, AST)	Yes (hepatic biopsy)	Yes	No	NAS improvement	No
Federico, 2019 [51]	(1) No treatment (30) vs. (2) silybin-phospholipid complex 606 mg + vitamin D 20 mg + vitamin E 30 mg (60)	Biopsy-proven NAFLD patients	RCT; 24 (on treatment) + 24 (wash-out; no treatment)	NA	NA	NA	NA	-	Higher percentage of patients with ALT and γ-GT decrease in group 2 (only in 6 months); higher percentage of patients with steatosis improvement in group 2 (TE)
Loguercio, 2012 [52]	(1) Placebo (69) vs. (2) silybin 188 mg + phosphatidylcholine 388 mg + vitamin E 179 mg (69)	Biopsy-proven NAFLD patients	RCT; 48	Yes (ALT, AST, γ-GT)	Yes (hepatic biopsy)	Yes	Yes	NAS improvement	γ-GT decrease in group 2
Athyros, 2006 [53]	(1) Atorvastatin 20 mg (63) vs. (2) fenofibrate 200 mg (62) vs. (3) atorvastatin 20 mg + fenofibrate 200 mg (61)	Non-diabetic NAFLD patients with MetS	Open-label, randomized; 54	Yes (ALT, AST, γ-GT)	Yes (US)	NA	NA	-	Higher percentage of patients with NAFLD resolution in groups 1 and 3 vs. group 2
Foster, 2011 [54]	(1) Placebo (36) vs. (2) atorvastatin 20 mg + vitamin E 1000 IU + vitamin C 1 g (44)	NAFLD patients	RCT; 192	NA	Yes (L/S ratio; CT)	NA	NA	-	Higher percentage of patients with NAFLD resolution in group 2
Della-Corte, 2016 [55]	(1) Placebo (23) vs. (2) DHA 500 mg + vitamin D 800 IU (18)	Biopsy-proven NAFLD children	RCT; 24 (on treatment) + 24 (wash-out; no treatment)	Yes (ALT)	Yes (hepatic biopsy)	Yes	No	NAS improvement	ALT decreased in group 2
Zöhrer, 2017 [56]	(1) Placebo (20) vs. (2) DHA 250 mg + choline 201 mg + vitamin E 39 IU (20)	Biopsy-proven NASH children	RCT; 48	Yes (ALT)	Yes (hepatic biopsy)	Yes	No	NAS improvement	NA
Polyzos, 2017 [57]	(1) Vitamin E 400 IU (17) vs. (2) vitamin E 400 IU + spironolactone 25 mg (14)	Biopsy-proven NAFLD patients	Open-label RCT; 52	No	Yes (NAFLD liver fat score)	NA	No (APRI)	-	No
Sanyal, 2004 [58]	(1) Vitamin E 400 IU (10) vs. (2) vitamin E 400 IU + pioglitazone 30 mg (10)	Non-diabetic, biopsy-proven NASH patients	RCT; 24	NA	Yes (hepatic biopsy)	Yes	Yes	-	Steatosis, ballooning and inflammation improved in group 2
Riche, 2014 [59]	Rosuvastatin 20 mg + pioglitazone 15 mg	NAFLD patients with obesity and T2DM	Case report; 36	Yes (ALT, AST)	Yes (US)	NA	NA	-	NA
Shah, 2011 [60]	(1) Insulin + placebo (13) vs. (2) insulin + pioglitazone 45 mg (12)	Patients with obesity and T2DM	RCT; 12–16	NA	No (L/S ratio; CT)	NA	NA	-	No
Zib, 2007 [61]	(1) Insulin (16) vs. (2) insulin + pioglitazone 30 mg (16)	Patients with T2DM	Open-label RCT; 24	No	Yes (MRS)	NA	NA	-	No
Torres, 2011 [62]	(1) Rosiglitazone 8 mg (31) vs. (2) rosiglitazone 8 mg + metformin 1000 mg (37) vs. (3) rosiglitazone 8 mg + losartan 50 mg (40)	Biopsy-proven NASH patients	Open-label RCT; 48	Yes (ALT, AST)	Yes, in the subgroup of patients with NASH (hepatic biopsy)	Yes, in the subgroup of patients with NASH	Yes, in the subgroup of patients with NASH	NAS improvement in the subgroup of patients with NASH	No
Omer, 2010 [63]	(1) Metformin 1700 mg (22) vs. (2) rosiglitazone 4 mg (20) vs. (3) metformin 1700 mg + rosiglitazone 4 mg (22)	Patients with NAS ≥ 5	Open-label RCT; 48	Yes (ALT, AST, γ-GT)	NA	NA	No (hepatic biopsy)	NAS improvement	NA
Lingvay, 2012 [64]	(1) Metformin 2000 mg + insulin (10) vs. (2) metformin 2000 mg + glyburide 2.5 mg + pioglitazone 45 mg (6)	Patients with T2DM (after a 3-month lead-in period of insulin + metformin treatment)	RCT; 124	NA	No (MRS)	NA	NA	-	No
Katoh, 2001 [65]	(1) Glibenclamide 3.7 ± 2.7 mg (38) vs. (2) glibenclamide 4.1 ± 2.5 mg + troglitazone 400 mg (40)	Patients with T2DM	RCT; 24	NA	NA	NA	NA	-	ALT, γ-GT decrease in group 2; steatosis improvement in group 2 (CT)
Sturm, 2009 [66]	(1) Diet (9) vs. (2) diet + metformin 1500 mg + pentoxifylline 12 mg (10)	Non-diabetic NASH patients	RCT; 48	No	No (hepatic biopsy)	NA	No	-	No
Sathyanarayana, 2011 [67]	(1) Pioglitazone 45 mg (10) vs. (2) pioglitazone 45 mg + exenatide 20 μg (11)	Patients with T2DM	Open-label RCT; 50	Yes (ALT, AST)	Yes (MRS)	NA	NA	-	ALT decrease in group 2; steatosis improvement in group 2
Shao, 2014 [68]	(1) Insulin aspart + insulin glargine (30) vs. (2) exenatide 10 μg (4 weeks) followed by 20 μg (8 weeks) + insulin glargine (30)	NAFLD patients with obesity and T2DM	RCT; 12	Yes (ALT, AST, γ-GT)	Yes (US)	NA	NA	-	ALT, AST, γ-GT decrease in group 2; higher percentage of NAFLD regression in group 2
Harreiter, 2021 [69]	(1) Placebo + dapagliflozin 10 mg (14) vs. (2) exenatide 2 mg + dapagliflozin 10 mg (16)	Patients with T2DM	RCT; 24	Yes (ALT, AST)	Yes (MRS)	NA	NA	Yes (FLI)	No
Gastaldelli, 2020 [70]	(1) Exenatide 2 mg + placebo (227) vs. (2) dapagliflozin 10 mg + placebo (230) vs. (3) exenatide 2 mg + dapagliflozin 10 mg (228)	Patients with T2DM	Post hoc of RCT; 52	Yes (ALT, γ-GT)	Yes (FLI and NAFLD liver fat score)	NA	Yes (NFS, FIB-4)	-	FLI and NAFLD liver fat score decrease in group 3 vs. group 1; ALT decrease in group 3 vs. group 1
Eriksson, 2018 [71]	(1) Placebo (20) vs. (2) omega-3 4 gr (15) vs. (3) dapagliflozin 10 mg (20) vs. (4) dapagliflozin 10 mg + omega-3 4 gr (20)	NAFLD patients with T2DM	RCT; 12	No	Yes (MRI-PDFF)	NA	NA	-	Steatosis improved in group 4 vs. group 1
Ku, 2021 [72]	(1) Metformin 2 gr + glimepiride ≥ 6 mg + DPP-4i + empagliflozin 25 mg (185) vs. (2) metformin 2 gr + glimepiride ≥ 6 mg + DPP-4i + dapagliflozin 10 mg (177)	Patients with T2DM	Open-label prospective observational; 144	NA	NA	NA	NA	-	No (LFTs)
Song, 2014 [73]	(1) Metformin 1500 mg + sitagliptin 100 mg vs. (2) metformin 1500 mg + glipizide 2.5–5 mg	NAFLD patients with T2DM	RCT; 16	Yes (ALT, AST, γ-GT)	NA	NA	NA	-	ALT, AST, γ-GT decrease in group 1; steatosis improved in group 1

^1^ Studies are sorted according to the sequence of their presentation in-text. Abbreviations: ALT—alanine aminotransferase; APRI—AST-to-platelet ratio index; AST—aspartate aminotransferase; CT—computed tomography; DHA—docosahexaenoic acid; DPP-4i—dipeptidyl peptidase-4 inhibitor; FIB-4—fibrosis-4; FLI—fatty liver index; LFTs—liver function tests; L/S ratio—liver-to-spleen attenuation ratio; MetS—metabolic syndrome; MRI-PDFF—magnetic resonance imaging–proton density fat fraction; MRS—magnetic resonance spectroscopy; NA—not available; NAFLD—nonalcoholic fatty liver disease; NAS—NAFLD activity score; NASH—nonalcoholic steatohepatitis; NFS—NAFLD fibrosis score; RCT—randomized controlled trial; T2DM—type 2 diabetes mellitus; TE—transient elastography; UDCA—ursodeoxycholic acid; US—ultrasound; γ-GT—γ-glutamyl transferase.

**Table 2:** Ongoing RCTs evaluating combination therapies in patients with NAFLD.

Medications; Date of Enrollment Initiation (Date/Month/Year) ^1^	Disease(s)	Estimated Enrollment (N)	Duration (Months)	Groups	Trial Identifier
Rosuvastatin and ezetimibe; 14 May 2018	NAFLD/Dyslipidemia	70	6	Rosuvastatin vs. rosuvastatin + ezetimibe	NCT03434613
Tropifexor and cenicriviroc; 11 September 2018	NASH	200	12	Tropifexor vs. cenicriviroc vs. tropifexor + cenicriviroc	NCT03517540
Pioglitazone and empagliflozin; 19 December 2018	NAFLD/T2DM	60	6	Pioglitazone vs. empagliflozin vs. pioglitazone + empagliflozin	NCT03646292
Garlic and silymarin and curcumin; 1 July 2019	NAFLD	60	3	Garlic + silymarin + curcumin vs. placebo	IRCT20190602043787N1
Tropifexor and licogliflozin; 11 December 2019	NASH	380	12	Tropifexor + licogliflozin vs. tropifexor + placebo vs. licogliflozin + placebo vs. placebo + placebo	NCT04065841
Saroglitazar and vitamin E; 16 December 2019	NAFLD	200	6	Saroglitazar vs. vitamin E vs. saroglitazar + vitamin E vs. lifestyle modifications	CTRI/2019/12/022339
Elobixibat and cholestyramine; 29 January 2020	NAFLD/NASH	100	4	Elobixibat + cholestyramine vs. elobixibat + placebo vs. placebo + cholestyramine vs. placebo + placebo	NCT04235205
LYS006 and tropifexor; 4 June 2020	NAFLD/NASH	250	5	LYS006 vs. LYS006 + tropifexor	NCT04147195
MET409 and empagliflozin; 15 December 2020	NASH/T2DM	120	3	MET409 vs. placebo vs. MET409 + empagliflozin vs. placebo + empagliflozin	NCT04702490
Empagliflozin and semaglutide; 26 March 2021	NAFLD/NASH/T2DM	192	12	Empagliflozin + semaglutide vs. empagliflozin + placebo vs. placebo + placebo	NCT04639414
Luseogliflozin and semaglutide; 29 July 2021	NASH/T2DM	60	12	Luseogliflozin + semaglutide vs. semaglutide	jRCTs061210009

^1^ Studies are sorted according the date of enrollment. Abbreviations: NAFLD—nonalcoholic fatty liver disease; NASH—nonalcoholic steatohepatitis; RCT—randomized clinical trials; T2DM—type 2 diabetes mellitus.

## Data Availability

Not applicable.
